# A Novel Approach to Improving Fat Delivery in Neonatal Enteral Feeding

**DOI:** 10.3390/nu7065051

**Published:** 2015-06-23

**Authors:** Jane Jarjour, Alexa M. Juarez, Denizen K. Kocak, Nathan J. Liu, Mika M. Tabata, Keli M. Hawthorne, Renata F. Ramos, Steven A. Abrams

**Affiliations:** 1 Department of Bioengineering, Rice University, Houston, TX 77005, USA; E-Mails: jane.jarjour@alumni.rice.edu (J.J.); juarezam@ymail.com (A.M.J.); Denizen.Kocak@utsouthwestern.edu (D.K.K.); tabata@alumni.rice.edu (M.M.T.); rfr1@rice.edu (R.F.R.); 2 Department of Medicine, Baylor College of Medicine, Houston, TX 77030, USA; 3 Beyond Traditional Borders, Rice University, Houston, TX 77005, USA; 4 Department of Medicine, University of Texas Southwestern Medical Center, Dallas, TX 75235, USA; 5 Department of Medicine, Imperial College London, London W12 0NN, UK; 6 School of Medicine, Stanford University, Stanford, CA 94305, USA; 7 Section of Neonatology, Department of Pediatrics, The University of Texas at Austin Dell Medical School, Austin, TX 77030, USA; E-Mails: keli.hawthorne@austin.utexas.edu (K.M.H.); sabrams@austin.utexas.edu (S.A.A.)

**Keywords:** enteral nutrition, breast milk, human milk-derived fortifier, very low birth weight, neonates, neonatal intensive care units, nutriflow

## Abstract

Continuous infusion systems used for enteral nutrition support in the neonatal intensive care unit deliver as little as 60% of the fat in human milk to the neonate. This study determined the effect of mixing common feedings for preterm infants in the feeding bag and tubing on fat losses during enteral feeding. Laboratory models were developed to assess the contribution of various mixing techniques to delivered fat content. Fat content was measured periodically during feeding and compared to baseline measurements. A multistage approach incorporating a feeding bag inverter and a tubing circulation loop delivered >90% of milk fat when used in conjunction with a commercial continuous infusion system. With unfortified human milk, this approach delivered 91.9% ± 1.5% of fat content over a one hour feed, significantly greater (*p* < 0.01) than 77.5% ± 2.2% delivered by continuous infusion controls (Mean ± SEM). With fortified human milk, this approach delivered 92.1% ± 2.4% of fat content, significantly greater (*p* < 0.01) than 79.4% ± 1.0% delivered by a non-adapted infusion system (Mean ± SEM). Mixing human milk during continuous infusion improves fat delivery, which may improve nutrition and growth outcomes in low birth weight neonates.

## 1. Introduction

Very low birth weight (VLBW) infants are commonly fed human milk enterally via gavage feeding [1,2,3]. Different methods of gavage feeding include intermittent gravity bolus and continuous infusion via a syringe or peristaltic pump. Despite the many advantages of feeding with human milk, slow growth rates in VLBW infants are common [2]. One factor which may contribute to this poor growth is the significant loss of fat from human milk during continuous infusion feeds, ranging from 37% to 47.5% [4,5,6,7,8]. Similar losses have been observed in human milk which has been supplemented with fortifier prior to feeding [9]. Since fat constitutes an important source of energy in feedings [6,10] and binds important mineral nutrients such as calcium and phosphorus in the milk [9], shortfalls in fat delivery to the infant present a clinically significant concern [2].

Fat loss during enteral delivery of human milk occurs when fat separates out from the aqueous milk components and adheres to the feeding bag and infusion set tubing [11,12]. Fortification of breast milk with human milk-derived fortifier and cream significantly improves fat delivery, but alternative, low-cost solutions have not yet been explored [13]. We hypothesized that mixing in the feeding bag and tubing could improve the nutrient delivery of continuous infusion pump systems. To test this hypothesis, we designed a three-component apparatus that operates in conjunction with the widely adopted Kangaroo ePump™ infusion system (Covidien, Mansfield, MA, USA), mixing human milk both in the feeding bag and tubing during feeds. In order to determine whether this approach significantly improved fat delivery compared to continuous infusion controls, we measured and compared fat content within the milk at the point of delivery over simulated one-hour feeds, both with and without use of the mixing apparatus.

## 2. Experimental Section

Laboratory *in vitro* models simulating enteral feeding in the NICU were designed for this study. In order to collect human milk for fat content analyses, the end of the feeding tube, which corresponds to the point of delivery to the neonate, was directed into collection containers. The feed rate used in the NICU environment corresponded to the collection rate in the laboratory setup.

### 2.1. Mixing Apparatus Design

In order to modify the existing Kangaroo ePump™ infusion sets, a three-component mixing device was built incorporating an inverter for the feeding bag, a circulation loop within the feeding tube, and microbore tubing. The feeding bag inverter and circulation loop operated throughout the simulated feed duration, both remotely programmed using an MSP430 microcontroller (Texas Instruments, Dallas, TX, USA).

**Figure 1:**
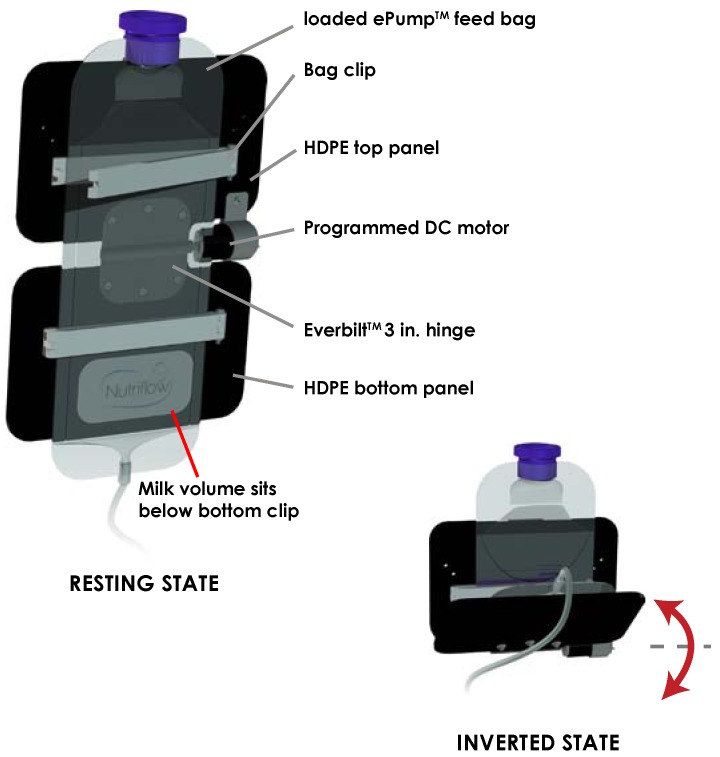
Schematic of inversion of milk volume from resting state to inverted state. Inversion of the ePump™ feeding bag occurs three times in quick succession every three minutes.

To address the separation of fat from aqueous milk in the feeding bag [11], a component was designed to invert the milk volume held in the feeding bag periodically throughout a feed as shown in Figure 1. The feeding bag inverter consisted of two panels of 0.125 inch high-density polyethylene (HDPE) plastic joined horizontally by a motorized hinge. A 5.5 inch bag clip (Linden Sweden Twixit, Edina, MN, USA) was fixed to the front of each panel. The top clip prevented the feeding bag from slipping, and the bottom clip reduced the surface area of fluid contact in the bag. The rear of the top panel was clamped to an IV pole, fixing the panel in a vertical orientation. Powered by a programmed 12 VDC motor acting upon the central hinge, the bottom panel rotated upwards 130 degrees from the vertical resting position, inverting the milk volume in the feeding bag. Following this motion, the motor switched off, and torque exerted by gravity returned the bottom panel and milk volume to its original resting orientation. Automated by an MSP430 microcontroller, the motor performed this inversion three times over ten seconds, followed by a three minute interval of rest before the next set of inversions.

To address fat separation and adherence in the ePump™ infusion set tubing, a circulation loop was designed to circulate fast-flowing milk through the tubing back to the feeding bag. A Y-shaped flow splitter (Quosina, Edgewood, NY, USA) was introduced at a point six inches away from the collection point, and a 20 inch segment of tubing ran from the flow splitter to the feeding bag. This created a path for milk to flow back to the feeding bag, controlled by a three-way solenoid pinch valve (Bio-Chem Fluidics, Boonton, NJ, USA). A second 12 VDC peristaltic dosing pump was used to drive faster flow during circulation periods. Two additional Y-shaped flow-splitters were inserted into the tubing three inches from each side of the existing Kangaroo ePump™. Connecting the pair of flow-splitting connections with a six-inch length of tubing allowed both the original Kangaroo ePump™ and the dosing pump to function in concert. For one minute out of every five minutes during collection, both the dosing pump and the three-way pinch valve switched on to simultaneously increase the flow rate (60 mL/min) and redirect fluid flow towards the feeding bag, cutting off all flow towards the collection point. For the remaining 4 min intervals, both remained off to allow normal flow (20 mL/h) towards the collection point. Precise timing control was accomplished using the MSP430 microcontroller, staggering schedules with the feeding bag inverter to prevent the introduction of air bubbles into the tubing that occurs when both processes run simultaneously.

Alteration of the diameter of the ePump™ infusion set tubing was also tested. In several feeds, the standard 3.0 mm inner-diameter tubing accompanying the Kangaroo ePump™ and used in the circulation loop was replaced with Sterilin™ microbore (0.5 mm ID) tubing (Thermo-Fisher Scientific, Waltham, MA, USA). This reduced the total milk volume contained in the tubing from 12 mL to 2.1 mL. Luer-lock and reducing connectors (Ibidi, Verona, WI, USA) allowed connection of the microbore tubing to non-adjustable components such as the feeding bag and Kangaroo ePump™.

The noise level of the device was measured with a dB-307 Noise Sound Level Meter (Toronto Surplus & Scientific Inc., Toronto, ON, Canada) in a quiet room. The noise level was 45.8 dB with the device turned off. With the motor on, the noise level was 54 dB, measured 36 inches from the device. When the device was circulating milk, the noise level was 59 dB. With both the motor on and milk circulating, the noise level was 59 dB.

### 2.2. Fat Content Testing

Human milk samples were obtained from five mothers of Neonatal Intensive Care Unit infants who had donated their milk for research purposes to the Mothers’ Milk Bank at Texas Children’s Hospital in Houston, Texas. All human milk samples were unprocessed, non-identifiable, and pooled together for this quality improvement study; there were no subject interactions with the research team. Therefore, Institutional Review Board approval was not required.

A total of 8 L of milk was aliquoted into 50 mL conical tubes. The milk was stored at −20 °C prior to use and was thawed to 25 °C within ten minutes from the start of each feed. Since human milk is often fortified with human milk fortifier (HMF) to address nutrient deficits in the premature infant [9,11,14,15,16], simulated feeds were also conducted where milk was fortified with the human milk fortifier H^2^MF Prolact+4^R^ fortifier (Prolacta, Monrovia, CA, USA) per manufacturer instructions. 100 mL of human milk or fortified human milk was swirled gently by hand and placed into the Kangaroo ePump™ feeding bag. Immediately prior to initiating the feed, a pre-infusion aliquot of 5 mL of milk was retrieved and analyzed for fat content.

Simulated feeds were conducted to measure delivered fat content when using the unaltered Kangaroo ePump™ continuous infusion system as a control case, or when coupling continuous infusion with one or more of the components described previously: (1) inversion of the feeding bag (I); (2) circulation of fast-flowing milk through the tubing (C); and (3) substitution of the standard pump tubing with 0.5 mm ID Sterilin™ microbore tubing (m). A summary of test conditions is provided in Table 1. Several mixing routines were combined during testing to assess the relative contribution of each combination to fat content. The Kangaroo ePump™ feeding bag was placed approximately one foot above containers used for final milk collection. Prior to initiating the feed, all pump tubing was filled with milk, following manufacturer instructions.

In our laboratory model, the infusion rate administered by the Kangaroo ePump™ was 20 mL/h. In addition to a pre-infusion aliquot (*t* = 0 min), milk was collected at 15 min intervals (*t* = 15, 30, 45, 60 min) over one-hour simulated feeds. Following collection, milk samples were stored at −20 °C before thawing for fat content analyses. Upon thawing to 25 °C, samples were subjected to sonication for ten seconds at 20 kHz (Q55 sonicator; Misonix Qsonica, Newton, CT, USA) before assessing fat content of milk using near-infrared spectrometry (Unity Scientific Spectrastar™ Neonatal Analyzer, Brookfield, CT, USA).

**Table 1:** Summary of Test Conditions.

Test Condition	Description
Bag inversion (I)	The Kangaroo ePump™ feeding bag was flipped automatically three times in quick succession every three minutes throughout a simulated feed.
Tube circulation (C)	An additional 20 inch segment of tubing which runs back into the feeding bag was connected 6 inches from the collection point. A second peristaltic pump was added to push milk flowing at a faster flow rate (15 mL/min) away from the neonate into this additional route back to the bag. Direction of flow was controlled using an automated three-way pinch valve. This fast flow circulation was automatically initiated for one minute out of every five minutes throughout a simulated feed.
Microbore tubing (m)	The original pump tubing (3.0 mm inner diameter) running between the feeding bag and the collection point (and circulation loop, if used in conjunction) was entirely substituted with 0.5 mm inner diameter Sterilin™ pump tubing, greatly reducing the milk volume that is held in the tubing.

### 2.3. Statistical Analysis

In order to account for variation in the pre-infusion fat content of the milk, the observed fat content of each feed sample (*t* = 15, 30, 45, 60 min) was normalized to that of the pre-infusion milk aliquot (*t* = 0 min), resulting in a percentage of fat content at each measurement. Statistically significant changes from the control case were calculated by performing Welch’s analysis of variance (ANOVA) test. Specifically, the endpoint (*t* = 60 min) measurement was highly valuable as it may suggest fat concentrations that could be delivered in simulated feeds of longer durations.

In order to obtain a single performance indicator of a method’s ability to deliver fat over the entire one-hour duration of the feed, area-under-the-curve (AUC) analysis was conducted to obtain an average efficiency of fat delivery. Since collection of milk volumes occurred over 15 min intervals, the measured fat concentration of each aliquot represented the mixed average delivered over the previous fifteen minutes of the feed. Therefore, the profile of fat concentration over the hour may be approximated by curves resembling a piecewise step function, using the fat concentrations measured at *t* = 15, 30, 45, and 60 min. By determining the AUC, a representation of the total delivered fat was obtained over the entire one hour duration of each feed. This value was normalized to the hypothetical amount of fat delivered if pre-infusion (*t* = 0 min) concentrations were maintained at the collection point over the entire hour, resulting in an efficiency indicator. Statistical analysis was conducted comparing each sample to controls using Welch’s analysis of variance (ANOVA) test.

## 3. Results

In total, 84 simulated feeds using pooled milk from five mothers were conducted. Conditions tested included inversion of the feeding bag, circulation of fast-flowing milk through the tubing, and substitution of the standard pump tubing with 0.5 mm inner diameter (ID) Sterilin™ microbore tubing. These conditions are summarized in Table 1, and hereafter referred to by the abbreviations I, C, and m, respectively. Each experimental setup was tested using a minimum of eight simulated feeds with unfortified human milk (Table 2). Additionally, four demonstrative experimental setups were tested using fortified human milk, using a minimum of three feeds each (Table 3).

**Figure 2:**
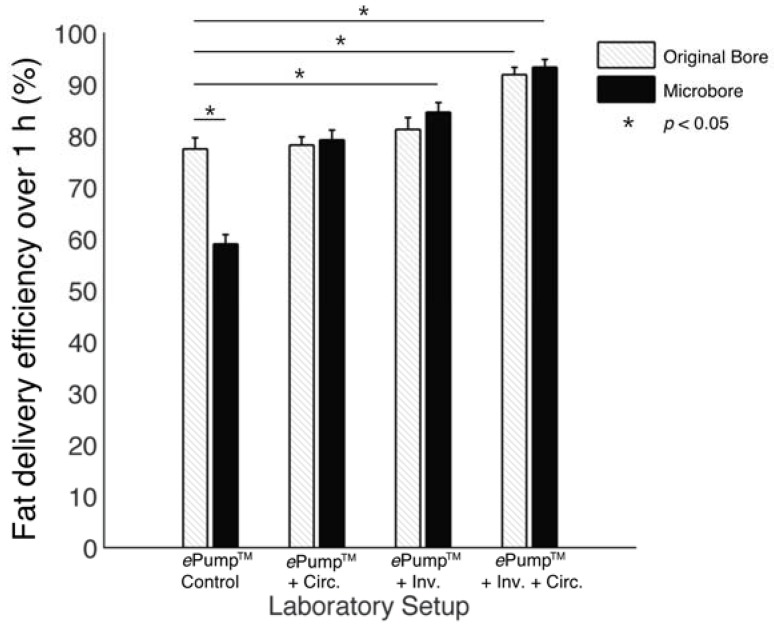
Average one-hour fat delivery efficiency in setups using unfortified breast milk. Test conditions using original pump tubing are shown in hatched bars, while test conditions using 0.5 mm ID microbore tubing are shown in black. Values are expressed as mean ± SEM of at least eight simulated feeds. *: significant change (*p* < 0.05) observed in delivery efficiency from the unmodified Kangaroo ePump™ infusion control.

The ePump™ + IC setup resulted in 91.9% ± 1.5% (± SE) fat delivery efficiency over one hour simulated feeds, a significant increase (*p* < 0.01) from the 77.5% ± 2.2% fat delivery efficiency of the control (Table 2, Figure 2). ePump™ + C feeds delivered 78.2% ± 1.6% and ePump™ + I feeds delivered 81.3% ± 2.3% of milk fat, neither of which presented a statistically significant increase over that of the control (Table 2, Figure 2).

**Table 2:** Fat content of unfortified milk.

Test	Kangaroo ePump™ (Control)	ePump™ + I	ePump™ + C	ePump™ + IC	ePump™ + m	ePump™ + mI	ePump™ + mC	ePump™ + mIC
# Simulated Feeds	8	8	8	8	8	8	8	10
Pre-infusion Fat Concentration (g/oz.)	3.5 ± 0.2	3.9 ± 0.3	4.0 ± 0.1	3.4 ± 0.3	3.1 ± 0.2	3.6 ± 0.4	3.6 ± 0.3	3.6 ± 0.3
Post-infusion fat concentration (g/oz.)	2.1 ± 0.1	3.1 ± 0.3	3.0 ± 0.2	3.1 ± 0.3	1.5 ± 0.1	3.0 ± 0.5	2.7 ± 0.1	3.3 ± 0.2
% decrease from pre-infusion levels at 1 h endpoint	40.58 ± 5.44	20.19 ± 6.70	26.22 ± 6.31	9.96 ± 4.30	50.41 ± 5.22	15.52 ± 5.95	25.82 ± 7.18	7.05 ± 6.70
Overall AUC fat delivery efficiency over 1 h (%)	77.46 ± 6.09	81.25 ± 6.45	78.21 ± 4.49	91.88 ± 4.10	59.01 ± 5.13	84.62 ± 5.17	79.25 ± 5.36	93.35 ± 4.78
Significant difference in fat delivery efficiency *vs.* control? (one-way ANOVA)	--	None (*p* = 0.12)	None (*p* = 0.39)	Increase (*p* < 0.01)	Decrease (*p* < 0.01)	Increase (*p* = 0.01)	None (*p* = 0.27)	Increase (*p* < 0.01)

**Table 3:** Fat content of milk fortified with H2MF Prolact + 4 Fortifier.

Test	Kangaroo ePump™ (Control)	ePump™ + IC	ePump™ + m	ePump™ + mIC
# Simulated Feeds	8	3	3	4
Pre-infusion (*t* = 0) fat Content (g)	4.6 ± 0.2	4.6 ± 0.1	4.4 ± 0.1	4.8 ± 0.3
Post-infusion (*t* = 60) fat content (g)	3.0 ± 0.3	4.3 ± 0.1	2.6 ± 0.1	4.3 ± 0.1
% decrease from pre-infusion fat content at 1 h endpoint	36.19 ± 7.56	9.21 ± 4.30	40.85 ± 3.62	8.87 ± 6.73
% total pre-infusion fat content delivered over 1 h (AUC)	79.38 ± 2.90	92.11 ± 4.12	70.11 ± 2.28	89.75 ± 4.98
Significant difference in total fat delivery *vs.* control? (one-way ANOVA)	--	Increase (*p* < 0.01)	Decrease (*p* < 0.01)	Increase (*p* < 0.01)

**Figure 3:**
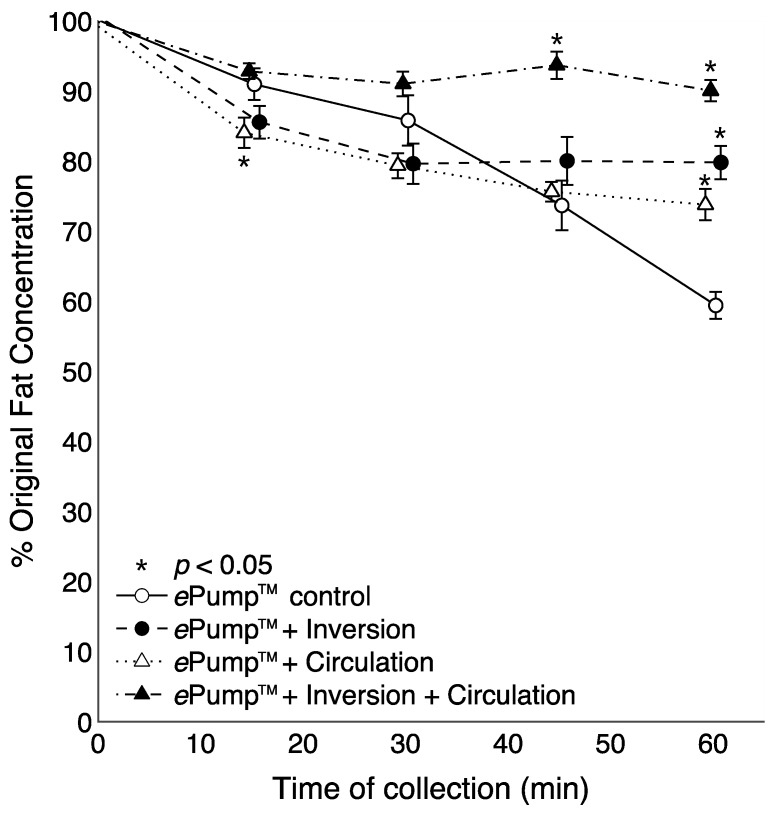
Changes in the percentage of pre-infusion (original) fat concentration of human milk delivered as a function of time. All tubing used had inner diameters (ID) of 3.0 mm as provided in the Kangaroo ePump™ set. Values are expressed as mean ± SEM of at least eight trials. *: significant change (*p* < 0.05) in percentage of original fat concentration at time point compared to Kangaroo ePump™ infusion control.

Figure 3 illustrates time-resolved fat concentrations measured in aliquots collected at 15 min intervals. All tests delivered significantly more fat than the control at the 60 min aliquot (*p* < 0.05), and the fat content delivered by the ePump™ + IC system was significantly greater than the control beginning with the 45 min aliquot (*p* < 0.05) (Figure 3).

### 3.1. Effects of Microbore Tubing

**Figure 4:**
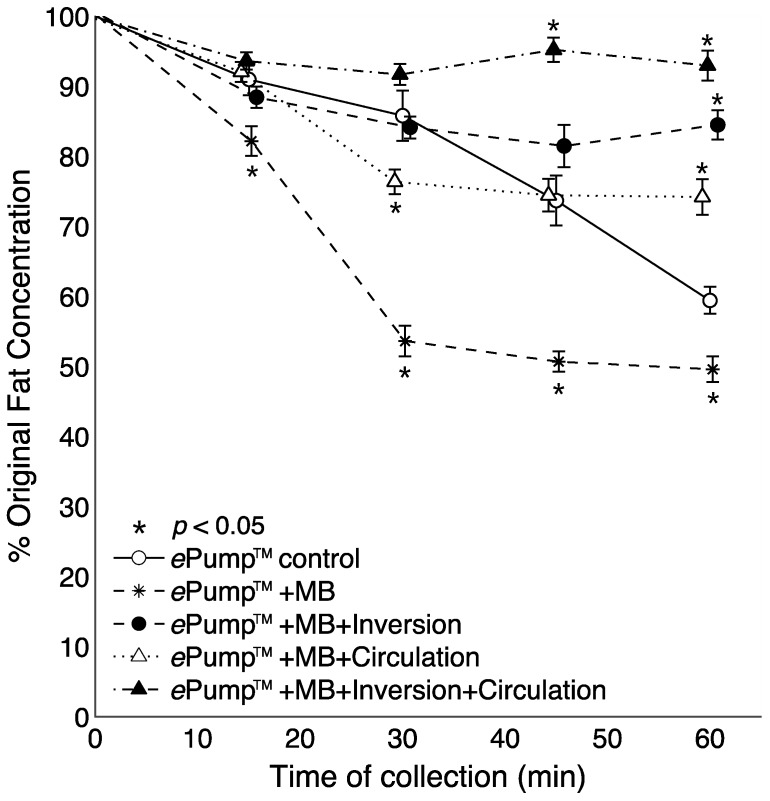
Changes in the percentage of pre-infusion fat concentration of human milk delivered as a function of time, incorporating setups using 0.5 mm ID microbore (MB) tubing. The milk samples were infused at 20 mL over one hour, with aliquots collected at 15 min intervals. Values are expressed as mean ± SEM of at least eight trials. *: significant change (*p* < 0.05) in percentage of original fat concentration at time point compared to Kangaroo ePump™ infusion control.

The impact of 0.5mm ID microbore tubing on fat delivery efficiency over a one-hour feed was also measured by replacing standard tubing with microbore tubing (Figure 4). The ePump™ + mIC system resulted in 93.4% ± 1.5% fat delivery efficiency, comparable to 91.9% ± 1.5% from the ePump™ + IC system using standard size tubing (Table 2, Figure 2). The ePump™ + mI setup resulted in 84.6% ± 1.8% efficiency, a significant increase over the control value, 77.5% ± 2.2% (*p* < 0.05), and comparable to the 81.3% ± 2.3% efficiency achieved by ePump™ + I. The ePump™ + mC feeds showed no significant difference compared to the control, and ePump™ + m feeds delivered only 59.0% ± 1.8% of milk fat, significantly less than the efficiency of the control (*p* < 0.05).

Figure 4 shows the time resolved data from feeds incorporating microbore tubing. All tests incorporating circulation or feeding bag inversion delivered significantly (*p* < 0.05) more fat than the control at the 60 min aliquot. The ePump™ + mIC system delivered significantly more fat starting at 45 min (*p* < 0.05). Use of microbore tubing with no mixing (ePump™ + m) resulted in significant decreases (*p* < 0.05) in fat concentrations compared to controls at all time points (Figure 4), also yielding a significant decrease in overall one-hour fat delivery efficiency (*p* < 0.05) (Figure 2).

### 3.2. Effects of Milk Fortification

**Figure 5:**
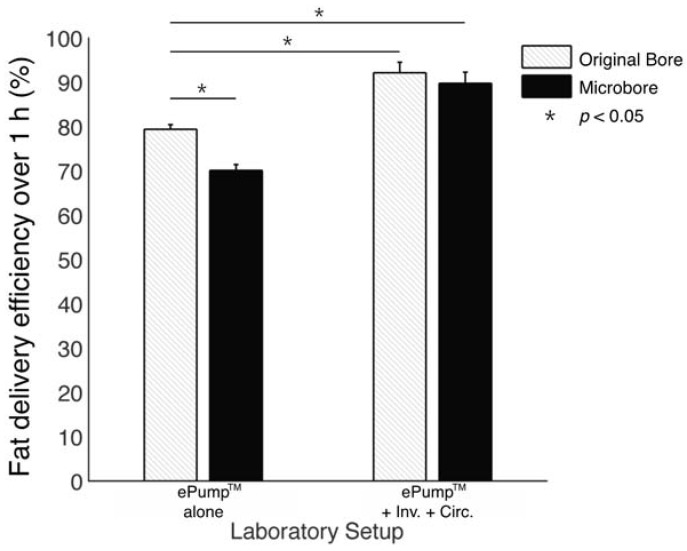
Average one-hour fat delivery efficiency in setups using fortified milk. Test conditions using original pump tubing are shown in hatched bars, while test conditions using 0.5 mm ID microbore tubing are shown in black. Values are expressed as mean ± SEM of at least three simulated feeds. *: significant change (*p* < 0.01) observed in fat delivery efficiency from the unmodified Kangaroo ePump™ continuous infusion.

Many tests were repeated using human milk fortified with H^2^MF Prolact+4^®^ fortifier. As indicated in Figure 5, the one hour fat delivery efficiency of both ePump™ + mIC (89.8% ± 2.5%, *p* < 0.05) and ePump™ + IC (92.1% ± 2.4%, *p* < 0.05) was significantly greater than the ePump™ control (79.38% ± 2.90%). The ePump™ + m tests resulted in a significant decrease in fat delivery efficiency (*p* < 0.05) compared to controls, delivering only 70.1% ± 1.3% of fat (Figure 5, Table 3).

**Figure 6:**
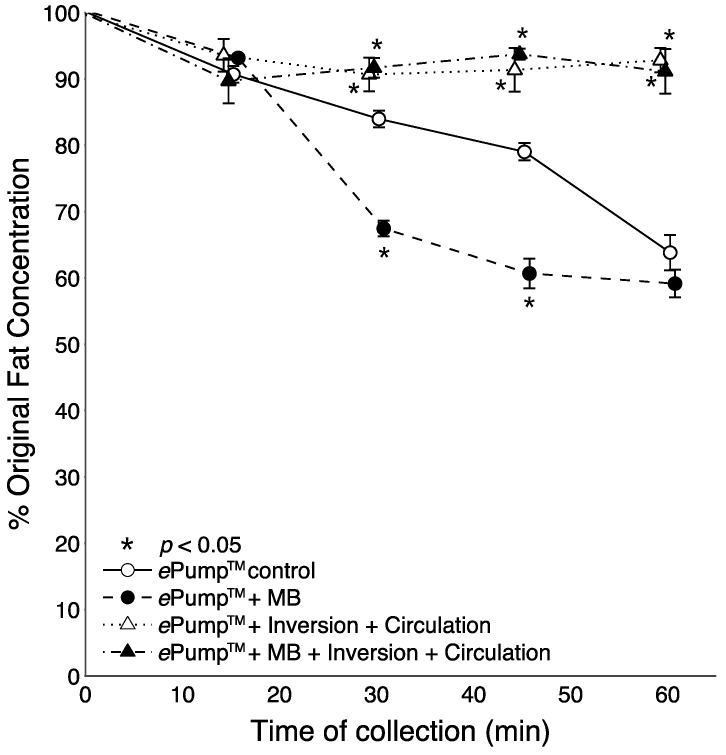
Changes in the percentage of pre-infusion fat concentration of fortified breast milk delivered as a function of time. Fortified milk samples infused at 20 mL over one hour, with aliquots collected at 15 min intervals. Values expressed as mean ± SEM of at least three trials. *: Significant change (*p* < 0.05) in percentage of original fat concentration at time point compared to Kangaroo ePump™ infusion control.

Fat content in milk aliquots taken at 15 min intervals were measured over the course of the feed using fortified milk as seen in Figure 6. Both the ePump™ + mIC and ePump™ + IC systems showed significant increases in fat delivery (*p* < 0.05) compared to the control beginning at the 30 min sample and continuing to the one hour endpoint (Figure 6). In contrast, the ePump™ + m setup showed significant decreases in fat delivery (*p* < 0.05) at 30 and 45 min time points.

## 4. Discussion

We have developed a novel approach to decrease fat losses in delivered human milk for high-risk infants. To our knowledge, this is the first practical apparatus that utilizes mechanical mixing methods to reduce fat loss during enteral feeding. While other approaches have been suggested to improve fat delivery during continuous infusion feeds, notably the addition of lecithin as an emulsifier [10] and the use of ultrasonication to pre-treat milk prior to feeding [12,17], unknown toxicity risks introduced by chemical additives and extensive efforts necessary for pre-treatment of milk volumes render these methods unrealistic for neonatal intensive care unit (NICU) implementation. Additionally, although the use of human milk-derived fortifier and cream improve fat delivery significantly [13], the mechanical mixing approach used in this study improves fat delivery to an even greater extent and is a low-cost solution.

Using an unmodified Kangaroo ePump™ continuous infusion system, we observed endpoint fat concentration reductions of approximately 40% from pre-infusion levels after one hour during simulated feeds of unfortified human milk. Similar reductions from pre-infusion levels were found in simulated feeds of fortified human milk. These results are consistent with fat losses during enteral feeding first reported three decades ago [5,6,7,9], indicating that infants may not be receiving prescribed energy intakes due to fat losses in the delivery system. Since enteral nutrition support using continuous infusion pumps is widely practiced in the NICU [3,18], our data indicates that nutrient losses during routine infant feedings remain a present concern.

Our design uses a strictly mechanical process to invert the feeding bag and circulate milk through the infusion set tubing to improve fat delivery. Our results demonstrate that a combination of the two processes significantly reduces both endpoint and overall fat losses during one-hour feeds, improving energy and nutrient delivery compared to the current standard of care. Agitation of the feeding bag has long been employed in the NICU, as nurses reported inverting Kangaroo ePump™ feeding bags after noticing fat accumulating at the milk surface. Through the use of microcontroller programming, our mixing apparatus automatically performs this action, allowing nurses to focus on other tasks.

Other design specifications facilitating NICU implementation include incorporation of bag clips for intuitive loading of milk volumes, as well as a peristaltic pump and solenoid pinch valves to control flow without directly contacting the milk, thereby minimizing contamination risk to the infant. The materials necessary to construct reusable components driving the inversion and circulation methods cost less than US $70, while alterations to the disposable Kangaroo feeding bag and tubing set add less than US $4 per use. The total noise level of the device in operation, 59 dB, is acceptable compared to other devices commonly used in the NICU, which range from 55 to 83 dB [19]. However, noise reduction would further improve the usability of the device.

Although inversion and circulation mixing processes significantly improved overall fat delivery when used simultaneously, only small improvements were seen when each was used individually. Since fat is lost both in the feeding bag and infusion set tubing, we postulate a synergistic effect results from the combination of inversion and circulation. Inverting the feeding bag resuspends milk fat to ensure that it enters the tubing, and circulation not only reduces the time milk must travel at slow flow rates, but also returns fat that has separated in the tubing to the bag for further mixing.

While increased fat delivery in continuous infusions using microbore tubing have been reported [8], our results demonstrate decreased fat delivery when microbore tubing is used independently of other mixing methods. Although the reason for this discrepancy is unclear, we speculate that a greater rate of fat loss occurs in the feeding bag than in the infusion set tubing. Microbore tubing effectually reduces the volume of milk held in the tubing, amplifying fat concentration changes in the feeding bag. This may explain our observation of increased fat delivery when microbore tubing is coupled with inversion of the feeding bag.

We did not evaluate fat delivery in feedings exceeding one hour, but our measured endpoint fat concentrations at one hour suggest potential benefits from our system in longer feeds. An additional limitation is the use of a single feeding rate of 20 mL/h. Increases in fat loss during feeding have been observed with decreased flow rates [6,7], and this suggests that milk mixing methods may be applied with greatest relative benefit at low flow rates, but is likely effective across the entire flow spectrum.

Future studies should modulate feed duration and flow rates to examine changes in relative improvement over unaided continuous feeds. Future studies should also evaluate fat loss when milk is fortified with bovine-based human milk fortifiers, as they are cheaper and more commonly used than the human milk-derived fortifier used in this study, and the additional acid in bovine-based fortifier may impact the total fat loss of a feed. Due to practical constraints, milk was frozen and thawed before use in this study, but there is evidence that the process of freezing and thawing might change the structural integrity of the milk fat globules [20,21]. Fresh milk (never frozen and never thawed) may result in further reductions in fat loss throughout a feed.

There is limited evidence correlating improved fat delivery with clinical outcomes. One studyobserved significant improvements in weight gain and anthropometric measurements of LBW infants fed sonication-treated human milk with improved fat content compared to infants fed unprocessed human milk [17]. Growth failure in LBW infants often arises from failure to ingest recommended energy intakes [22,23], which are hindered by maximum tolerated flow rates of milk [18]. Enhancing nutrient delivery by mixing milk during infusion will allow for improved energy intake within tolerable flow rates. This may result in enhanced weight gain and improved feeding tolerance, enabling earlier transitions to preferred gravity (bolus) infusions. Future studies are necessary to directly correlate improved fat delivery to clinical outcomes in LBW infants, such as weight and length gain. Our mixing device is a suitable apparatus for achieving increased fat content in order to collect such measures.

## 5. Conclusions

Fat losses from currently used continuous infusion pumps impact the growth and development of VLBW infants. We have demonstrated proof of concept that mechanical mixing in both the feeding bag and tubing can improve fat delivery of human milk in neonatal enteral feeding. Our prototype device for automated mixing, incorporating a feeding bag inverter and a tubing circulation loop, delivered >90% of milk fat when used in conjunction with a commercial continuous infusion system. When delivering both unfortified human milk and fortified human milk, mechanically mixing milk with our device showed significant improvement in fat delivery compared to fat delivery by a non-adapted infusion system. Further development, commercialization, and clinical testing of this device and concept may lead to improved nutrition and growth outcomes in VLBW infants.
